# A Review of Acute Aerobic Exercise and Transcranial Direct Current Stimulation Effects on Cognitive Functions and Their Potential Synergies

**DOI:** 10.3389/fnhum.2018.00534

**Published:** 2019-01-11

**Authors:** Fabian Steinberg, Nils Henrik Pixa, Felipe Fregni

**Affiliations:** ^1^Institute of Sport Science, Johannes Gutenberg-University Mainz, Mainz, Germany; ^2^Sport Psychology, Institute of Human Movement Science and Health, Faculty of Behavioral and Social Sciences, Chemnitz University of Technology, Chemnitz, Germany; ^3^Spaulding Neuromodulation Center, Spaulding Rehabilitation Hospital and Massachusetts General Hospital, Harvard Medical School, Boston, MA, United States

**Keywords:** non-invasive brain stimulation, neuro-rehabilitation, cognitive training, transcranial electric stimulation, executive functions, physical activity, cognitive enhancement, tDCS

## Abstract

Today, several pharmaceutic and non-pharmaceutic approaches exist to treat psychiatric and neurological diseases. Because of the lack of treatment procedures that are medication free and without severe side effects, transcranial direct current stimulation (tDCS) and aerobic exercise (AE) have been tested to explore the potential for initiating and modulating neuroplasticity in the human brain. Both tDCS and AE could support cognition and behavior in the clinical and non-clinical context to improve the recovery process within neurological or psychiatric conditions or to increase performance. As these techniques still lack meaningful effects, although they provide multiple beneficial opportunities within disease and health applications, there is emerging interest to find improved tDCS and AE protocols. Since multimodal approaches could provoke synergetic effects, a few recent studies have begun to combine tDCS and AE within different settings such as in cognitive training in health or for treatment purposes within clinical settings, all of which show superior effects compared to single technique applications. The beneficial outcomes of both techniques depend on several parameters and the understanding of neural mechanisms that are not yet fully understood. Recent studies have begun to directly combine tDCS and AE within one session, although their interactions on the behavioral, neurophysiological and neurochemical levels are entirely unclear. Therefore, this review: (a) provides an overview of acute behavioral, neurophysiological, and neurochemical effects that both techniques provoke within only one single application in isolation; (b) gives an overview regarding the mechanistic pathways; and (c) discusses potential interactions and synergies between tDCS and AE that might be provoked when directly combining both techniques. From this literature review focusing primarily on the cognitive domain in term of specific executive functions (EFs; inhibition, updating, and switching), it is concluded that a direct combination of tDCS and AE provides multiple beneficial opportunities for synergistic effects. A combination could be useful within non-clinical settings in health and for treating several psychiatric and neurologic conditions. However, there is a lack of research and there are several possibly interacting moderating parameters that must be considered and more importantly must be systematically investigated in the future.

## Introduction

One of the most striking characteristics of the human brain is its ability to respond to changing internal and external states by reorganizing and restructuring neural circuitry on different timescales ranging from milliseconds to many years. This ability, called neuroplasticity, is extremely important throughout a person’s entire life, whether it is in skill or knowledge acquisition or within recovery processes after brain injury (Kays et al., 2012). Synaptic strengthening and building new synaptic connections is a key element within neuroplasticity and must be studied to determine the best means to recover or initiate optimal neuroplastic processes in case of disruptive events through disease or injuries that provoke maladaptive brain functions and abnormal neuroplasticity (Nitsche et al., 2012). Traditional and conventional treatments of maladaptive brain functions and neuroplasticity include medications; however, these are often accompanied by severe side-effects (De Hert et al., 2011). Therefore, there is an increasing need for non-pharmacological, cost-effective, harmless, and easily applicable, yet still effective treatments that can be used to replace drugs or as supplements to increase overall treatment success. Two techniques are of relevance to fulfill such needs: transcranial current stimulation (tCS), such as transcranial direct current stimulation (tDCS), and aerobic exercise (AE).

The tDCS technique provides a non-invasive and safe way to modulate neuronal activity and provoke neuroplastic changes, which has been predominantly shown in the human motor cortex, such as the primary motor cortex (M1) (Huang et al., 2017). tDCS applies a weak and constant current via surface electrodes attached to the scalp and placed over a target brain area. While most tDCS studies stimulated motor regions, several works have been applied to investigate the effects of tDCS on non-motor regions, such as the prefrontal cortex (PFC). In particular, prefrontal tDCS is a promising technique of initiating neural plasticity within neurological and psychiatric conditions that are associated with maladaptive neuroplasticity (for a review see Flöel, 2014). Different forms of regular AE have also been associated with functional and structural brain adaptations enabling the individual to better adapt to new demands (Hötting and Röder, 2013). Moreover, the research of acute effects of AE have documented a series of effects on the cognitive domain in health and disease (McMorris, 2016b). These techniques provide promising and multiple beneficial opportunities in neurologic and psychiatric diseases (Fregni et al., 2006a; Knöchel et al., 2012; Kuo et al., 2014; Forbes et al., 2015) and also in health (Choe et al., 2016; Ward et al., 2017). More specifically, there is increasing evidence from clinical trials that both tDCS and AE can be beneficial in stroke (Duncan et al., 2003; Fregni et al., 2005; Tang et al., 2009; Brunoni et al., 2012; Marquez et al., 2015), fibromyalgia (Castillo-Saavedra et al., 2016; Fink and Lewis, 2017), Alzheimer’s disease (Ferrucci et al., 2008; Intlekofer and Cotman, 2013; Farina et al., 2014; Hsu et al., 2015; Inagawa et al., 2018), Parkinson’s disease (Fregni et al., 2006c; Schenkman et al., 2012), major depressive disorder (Fregni et al., 2006b; Kuo et al., 2014; Schuch et al., 2016; Yokoi et al., 2018), and schizophrenia treatments (Gorczynski and Faulkner, 2010; Kuo et al., 2014; Smith et al., 2015; Yokoi et al., 2018).

However, despite such promising results, clinical effectiveness is not yet fully supported as both techniques often lack meaningful effect sizes (Kekic et al., 2016). Therefore, recent research has explored optimal exercise protocols such as the ideal dosage and modalities. Also, optimal tDCS stimulation protocols, e.g., electric intensity, electrode configuration, and duration, were studied (Paulus, 2011; Woods et al., 2016). Moreover, improved techniques such as optimized focality (Datta et al., 2009), multi-electrode (Dmochowski et al., 2011; Pixa et al., 2017a,b), brain priming (Christova et al., 2015; Hurley and Machado, 2017), or network stimulation (Fischer et al., 2017) were used to improve tDCS effects.

Other researchers have proposed that multimodal approaches (i.e., more than one intervention technique) may initiate synergistic or additive effects and increase effectiveness (Ward et al., 2017; Cespón et al., 2018). Multimodal approaches can only be effective if supposed mechanistic pathways of both to-be-combined techniques justify an additive outcome with respect to the targeted system. Recent works took advantage of the exclusive and converging mechanistic pathways of tDCS and AE, including their possibilities to initiate cortical plasticity processes. Those possibilities presented theoretical considerations (Moreau et al., 2015) and promising empirical findings in the combined use of tDCS and AE in therapeutic, non-clinical, and sports settings (Okano et al., 2015; Angius et al., 2017; Edwards et al., 2017; Ward et al., 2017). In a randomized control trial (RCT), tDCS and AE resulted in greater improvements in multiple cognitive domains in a 4-month cognitive training intervention in healthy persons more than each technique alone, indicating promising opportunities for complex occupational settings (Ward et al., 2017). Moreover, combined tDCS/AE applications in a RCT reduced pain perception in fibromyalgia (Mendonca et al., 2016) and reduced appetite sensation in a single session experiment (Montenegro et al., 2012). Other directly sport-related studies were also able to modulate perceived exertion in a submaximal cycling exercise task (Okano et al., 2015). In addition, improved endurance performance was found (Angius et al., 2017, 2018; Edwards et al., 2017). Based on this potential of tDCS to improve sport performance, potential applications in sport-related skills are currently discussed (Colzato et al., 2017).

When reviewing the new and multimodal empirical studies, the rationale for a combination of tDCS and AE and their potentially targeted mechanisms, which are responsible for desired additive/synergistic effects, often remained vague and on a superficial level. This lack of knowledge may be because: (a) the exact mechanistic pathways of tDCS and AE in exerting their impact on the brain are still not fully understood; (b) there is a large number of moderating parameters and variabilities known for each technique alone; and (c) there is a complete lack of knowledge about how those moderating parameters interact specifically and how both techniques interact generally when applied together. This work describes the mechanistic pathways of tDCS and AE and asserts that understanding the possible interactions of these pathways is critical when designing combination interventions aimed at improving cognition in healthy individuals or patients suffering neuropsychiatric or neurological diseases.

Based on chronic exercise effects (i.e., the effects of exercise training for several weeks or months), Moreau et al. (2015) provide a description and rationales of mechanistic pathways (2015) for tDCS/AE, and Hendrikse et al. (2017) also discussed the effects of the combination of AE with repetitive transcranial magnetic stimulation (rTMS). Acute effects and their possible interactions provoked by tDCS and AE methods on behavioral, neurophysiological and neurochemical levels even within a single application and short time succession have not been systematically addressed when combining tDCS and AE (Moreau et al., 2015; Hendrikse et al., 2017). This article reviews the reports of acute effects (specifically, only one application) in the tDCS and AE field, and the effects that should be carefully considered when combining both methods for future research or clinical use. Due to the large variety of effects on several brain functions and behaviors, the primary focus is on the acute effects of AE and tDCS on cognitive abilities and particularly on executive functions (EFs).

Our literature search in central databases (i.e., MEDLINE, Web of Science, PsycINFO and Google Scholar) for this narrative review primarily included studies that investigated the effects of AE (i.e., no resistance exercise) or tDCS (i.e., no other brain stimulation method) on tests that measure EFs or cognition in a single session intervention. Based on this search we also checked the reference list of selected articles. The intention of such a broad search strategy was to find as many studies as possible, simultaneously reducing the risk of missing any studies that combined tDCS and AE in one single session intervention on the cognitive system. We used the logical operators “OR” and “AND” between exercise-related terms (i.e., exercise and physical activity) and brain stimulation terms (tDCS and transcranial direct current stimulation) and the cognition search modifier cogniti* (i.e., cognition, cognitive). We did not restrict the time interval of the search but concentrated our study description on tasks that are thought to measure EFs (see section “Executive Functions and Their Neuroanatomical Basis”). We also excluded studies that investigated effects in more than one session (e.g., chronic exercise effects on cognitive functions or tDCS repetition across days) and in other domains (e.g., the motor or emotional domain) as this would require additional reviews. Based on this review and the performed comparison of tDCS and AE effects on EFs, possible interacting pathways and mechanisms are discussed while additionally consulting insights from studies focusing on other functions (e.g., motor domain). As there are several forms of exercise paradigms, we use the term AE when cardiovascular exercise had been performed within highly automated movements such as in cycling, walking or running. AE includes physiological changes on the metabolic, respiratory and cardiovascular system of the body, while anaerobic exercise includes higher intensity activities which can be maintained only during a short time frame. Other exercise paradigms are resistance exercise (i.e., strength training) which affects also metabolic systems but also intra- and inter-muscular coordination, or coordinative exercise which often involve less physiological demand on the human body (Voelcker-Rehage and Niemann, 2013; Voelcker-Rehage et al., 2017). This review primarily focuses those effects that are elicited by AE protocols, while indicating whether another paradigm has been applied in a specific study.

### Executive Functions and Their Neuroanatomical Basis

Early researchers in the field of EFs described a “central executive,” (Baddeley and Hitch, 1974) or a “supervisory attentional control system” (Norman and Shallice, 1986). Both models propose a specific kind of a superordinate control instance that acts as the central mechanism which coordinates and processes higher cognitive functions. Despite controversy about exact definitions and cognitive models, there is a consensus about the complexity of EFs and significance to human adaptive behavior (Jurado and Rosselli, 2007). Today, it is widely accepted that EFs are an umbrella term for a set of lower-level cognitive processes that serve higher cognitive processes such as self-regulation, coping with novel situations, complex planning, and decision making (Miyake et al., 2000; Friedman et al., 2008; Miyake and Friedman, 2012; Niendam et al., 2012; Diamond, 2013). One key question that remained unresolved was whether there is one single executive functioning (unity) or whether there are distinct functions (diversity) (Jurado and Rosselli, 2007). Current influential assumptions in cognitive psychology and neuroscience provide evidence for both perspectives and indicate that EFs are unitary and non-unitary in nature (e.g., Duncan et al., 1996; Godefroy et al., 1999). Based on confirmatory factor analysis, Miyake et al. (2000) stated that the three following unique, but not completely separable, EFs form the core aspects of cognitive control: updating relevant information in the working memory (i.e., updating ability), switching between different tasks and rule sets (i.e., shifting ability), and inhibiting responses to dominant, pre-potent stimuli (i.e., inhibition ability; Miyake et al., 2000).

More generally, EFs are mainly processed in a superordinate, widespread frontal–cingulate–parietal–subcortical cognitive control network of the brain (Niendam et al., 2012). Thus, activations of different brain areas are integrated to guide behavior, attention regulation, thought, goal setting, and other higher-level cognitive abilities (Alvarez and Emory, 2006; Jurado and Rosselli, 2007). Despite the fundamental necessity of the integrity of the entire brain in forming controlled behavior, the PFC is of exceptional relevance as this brain area and its sub regions orchestrate behavior by integrating information coming from other cortical and subcortical areas (Stuss and Alexander, 2000; Stuss et al., 2002). Such involvement of the PFC in EFs has been identified in neuropsychological studies on human patients with lesions in PFC structures by neuroimaging studies and also in various animal studies (Stuss and Alexander, 2000; Stuss et al., 2002). Brain lesions in the PFC provoke decreased performance in various tasks requiring EF abilities, including flexibility, working memory, and inhibition (Niendam et al., 2012), indicating that the intact function of this area is fundamental for optimal performance (Stuss and Alexander, 2000).

Similar brain activation patterns are observable across EF tasks, but there is also a unique and specific activation pattern within single EFs (Niendam et al., 2012). Experimental evidence exists that EFs are processed by various interconnected brain regions, ranging from frontal and motor areas to subcortical structures (Niendam et al., 2012). More specifically, the dorsolateral PFC (DLPFC), medial frontal cortex, anterior cingulate cortex (ACC), frontal and posterior parietal cortex, motor areas, and cerebellum are all involved in EF processing (Fuster, 2002; Badre and D’Esposito, 2007; Bellebaum and Daum, 2007; D’Esposito, 2007). Further analyses and meta-analysis of brain areas activated between EFs (i.e., domain-specific areas) indicate different activity patterns in the anterior PFC, anterior and midcingulate regions, and even in unique subcortical regions such as the basal ganglia, cerebellum and thalamic pathways (Kassubek et al., 2004; Lewis et al., 2004; Monchi et al., 2006; Niendam et al., 2012). Thus, those differences in neural activation patterns across cognitive processing together with the coactivation of common structures during cognitive task support the unity-diversity perspective that was proposed based on behavioral data (Miyake et al., 2000; Miyake and Friedman, 2012; Snyder et al., 2015).

### Executive Function Tests

Many test procedures exist that have been developed to test various EFs. Some of the more complex tests [e.g., the Tower of London (TOL), Wisconsin Card Sorting Test] tap into multiple EFs or have been used in one study to test one specific EF and in another to test a different EF. For example, the well-known Stroop test has been used in AE studies to measure inhibition ability in one study (Peruyero et al., 2017), but it is often used or termed as being a measure of cognitive flexibility (Masley et al., 2009). This non-specificity, lack of definition and terminology across studies has been criticized in some influential models; thus, it is often difficult to clearly state which test measures a specific ability (Miyake et al., 2000; Miyake and Friedman, 2012; Snyder et al., 2015). Nevertheless, according to recent works, the updating ability represents a specific kind of working memory that has often been tested by the N-back, Keep track, Sternberg and other, more complex neuropsychological tests (Snyder et al., 2015). This function is concerned with storing a specific amount of information in working memory while this information has to be continuously updated with new incoming information; in other words, irrelevant “old” information must be removed from working memory and new information must be stored and handled mentally. Typical performance parameters include reaction time and accuracy. The shifting function is responsible for applying a new task rule that must be fulfilled. Specifically, the shifting function controls the flexible switch from one concept to the other based on task demands. Tests to measure the switching ability are, for example, the Global-local, Trail-making or the Number-letter tasks. Typical performance parameters include the switching costs, which can be calculated by the differences in reaction times between stimuli (within a test) where no switch was necessary and with those stimuli preceding a rule switch (i.e., the score represents the time for rule shifting). The inhibition function is thought to control the correct identification of relevant task stimuli while ignoring task-irrelevant, yet pre-potent stimuli. In other words, the inhibition function ensures that responses to task-irrelevant stimuli must be inhibited. Some of the most common tests are the Wisconsin Card Sorting, Stroop, Flanker, Simon, Stop-signal, or Go/no-go tests.

## Aerobic Exercise Effects on Executive Functions

Early and systematic investigations on the acute exercise-cognition interaction observed that several cognitive abilities can be modulated (positively and negatively) by a short period of physical whole-body exercise (McMorris, 2016c). Typical study designs include a baseline measure of the cognitive function of interest with a subsequent AE intervention and a retesting of the cognitive function. Retesting was performed either during or after the AE intervention with the focus of online or offline effects, respectively. Usually, a control condition was included where either the same (cross-over) or another participant group (between-design) performed the same tests in the same sequence in rest or in another activity. Thereby, exercise was traditionally defined as being a stressor; thus, its interaction with cognition would follow an inverted-U profile derived from the Yerkes-Dodson arousal-performance theory (see McMorris and Hale, 2012 for an overview). This theory posits the existence of an optimal relationship between the arousal level and cognitive performance (Yerkes and Dodson, 1908). As long as arousal is on a “too low” or “too high” level and not at the peak of the inverted-U, it means that optimal cognitive performance cannot be achieved without any change in the arousal level. This model was transferred to the AE field, suggesting that physical exercise modulates the arousal level based on the intensity (see further below on mechanistic pathways of how AE modulates arousal).

One limitation of this access is that exercise intensity definitions have varied across research on the exercise-cognition relationship, leading to inconsistent definitions and consequently heterogeneous findings. However, several exercise intensity definitions exist when describing relative or absolute physiological or subjective parameters (Norton et al., 2010; American College of Sports Medicine, 2018). Relative indices include parameters such as the individual heart rate ranges (i.e., % of maximal heart rate), oxygen uptake (i.e., % of maximum oxygen uptake = VO_2_max), and ratings of perceived exertion scales (e.g., range between 6 = very low and 20 = absolute limit). Absolute intensity metrics include parameters such as the metabolic equivalent (MET). One current categorization (American College of Sports Medicine, 2018) of exercise intensity, such as the % of maximal heart rate (%HRmax), includes five distinct exercise intensities: inactive below 50%, low between 50% and 64%, moderate between 65% and 74%, high (or vigorous) above 75% and maximal at 100%. Based on those or comparable categorizations, several studies have hypothesized that as exercise intensity is low (low arousal), the performance in various cognitive tasks is low (including EF tasks using the Stroop, Flanker or more complex neuropsychological tests such as the TOL test and including memory, attention, and choice-reaction time tasks); in addition as exercise intensity rises to moderate levels, performance (and arousal) increases. Subsequently, the cognitive performance decreases again with rising exercise intensity levels (McMorris, 2016c). However, less empirical evidence supports the inverted-U hypothesis, and various reviews and meta-analyses are inconsistent and even provide opposite conclusions (Brisswalter et al., 2002; Tomporowski, 2003; Lambourne and Tomporowski, 2010; McMorris et al., 2011; Chang et al., 2012; McMorris and Hale, 2012, 2015). Specifically, cognitive performance was not consistently associated with intensity levels following an inverted-U profile (e.g., Chang et al., 2012).

Heterogeneous findings are due to many multifaceted and interacting parameters that can moderate the exercise-cognition interaction. It has been extensively shown that exercise intensity can influence the performance of EFs (Kamijo et al., 2007) and cognition in general (Chang et al., 2012). Labelle et al. (2013), as an example, found that the accuracy scores in a Stroop test declined during a high intensity AE cycling task compared to moderate intensity. It has also been repeatedly shown that the timing of cognitive test administration can affect the outcome of the cognitive test. According to a meta-analysis across different cognitive domains by Chang et al. (2012), no effects on cognitive functions occurred within the first 10 min of exercise, negative effects emerged between 11 min and 20 min of exercise, and positive effects appeared after 20 min (Chang et al., 2012). Such a conclusion has been confirmed for the EF inhibition ability (reaction time of incompatible stimuli in a Stroop test), as 20 min of moderate exercise intensity improved inhibition, while 10 and 45 min had no effects on inhibition (Chang et al., 2015b). The Chang et al. (2012) meta-analysis also showed positive effects on cognitive functions regardless whether cognitive tests were administered during exercise, immediately following exercise, or after a time delay. Another meta-analysis observed a small negative effect of cognitive performance during AE (Lambourne and Tomporowski, 2010). This discrepancy potentially occurred because the latter study focused only on healthy participants and crossover designs (see Chang et al., 2012).

Also, exercise modality has an effect on EFs since Pontifex et al. (2009) showed that reaction times in a Sternberg task (working memory) were faster (compared to rest condition) after only 30 min of AE and not after a bout of resistance exercise of 30 min. Interestingly, even a change in environmental factors (e.g., changed gravity or confinement) affected cognitive functions differently compared to AE conditions (Schneider et al., 2013; Vogt et al., 2014). Other studies have shown that the exercise-cognition relationship can be modulated by cognitive task difficulty, the cognitive domain (i.e., whether EFs or other cognitive tasks relating to attention or pure memory task are measured), and age (Kamijo et al., 2009; Weng et al., 2015; Voelcker-Rehage et al., 2017). Despite the age and cognitive domain, a further crucial factor seems to be the individual fitness status (Labelle et al., 2013). During exercise (i.e., online), individual fitness level is associated with enhanced cognition for highly fit subjects, but is negligible in moderately fit and decreased for unfit participants; while only unfit and highly fit, but not moderately fit participants benefited after exercise (i.e., offline) (Chang et al., 2012). A recent systematic review focusing on high-intensity exercise in trained people found that acute effects are dependent on the cognitive domain. In 10 reviewed studies simple tasks were not affected while the effects were stronger in parameters indicating speed of processing compared to accuracy parameters in complex tasks (Browne et al., 2017).

Several meta-analyses and reviews, however, suggest that EFs, within the broad domain of cognition, benefit from AE regardless of exercise paradigm, modality, intensity and time of testing (Chang and Etnier, 2009; Lambourne and Tomporowski, 2010; Chang et al., 2012, 2015a,b). In addition, it is argued that moderate exercise intensity is most beneficial; however, these positive effects in cognitive tasks have been mostly found in terms of speed of processing (across cognitive domains) and not accuracy (e.g., the number of errors made in a specific test), while the observed effect sizes were highest for EFs compared to tasks of alertness/attention and recall (McMorris and Hale, 2012).

Moreover, moderate exercise sessions affect speed of processing positively and accuracy slightly negatively, especially in working memory tasks (e.g., in N-back tests) (McMorris et al., 2011; McMorris and Hale, 2012). However, in this meta-analysis, the inclusion criteria of working memory tasks included all EF tasks (e.g., Stroop and flanker tasks), thus not agreeing with the discussed definition (e.g., a Stroop test does not assess working memory performance). In contrast, recent studies, such as Tempest et al. (2017), show that performing high-intensity exercise improved inhibition performance (reaction time in a flanker test) but decreased updating performance (aggravated d’ value of a 2-back test; d’ value represents task performance accounting for accuracy and reaction time) suggesting that EFs are not uniformly affected by exercise, despite having the same test protocol (Weng et al., 2015; Tempest et al., 2017). These different effects indicate that exercise selectively affects neural networks and possibly prefrontal sub-regions that support the different EF abilities. However, whether this is due to time or intensity dependent properties of exercise and thus due to differences in methodological study designs remains an open question. Furthermore, caution is needed as most evidence for the acute exercise effects on EFs have been derived based on EF tests mainly testing inhibition (Ludyga et al., 2016), and there are also several studies suggesting no effects or even detrimental effects (Basso and Suzuki, 2017).

## tDCS Effects on Executive Functions

Due to the significant involvement of the PFC in cognitive processing and in EFs, a growing amount of studies have analyzed tDCS-induced modulations of PFC activity and its possibility to enhance cognitive functions including EFs (see Strobach and Antonenko, 2017) in the short-term (see Tremblay et al., 2014) and in the long term (Park et al., 2014; Metzuyanim-Gorlick and Mashal, 2016). The typical study design for analyzing acute tDCS effects on EF ability or other cognitive domains includes a pretest of the cognitive function of interest, followed by an intervention, where the cognitive test is performed during (i.e., online effects) or after (i.e., offline effects) tDCS. Usually, the same test sequence was performed either in a cross-over or between-subject design, where a sham stimulation (i.e., placebo) was provided and/or real tDCS at a brain region suspected not to be involved in cognitive processing (e.g., M1). If applicable, the experiment was performed with a single-blind (participant was not aware whether stimulation is real or sham) or at best cases, with a double-blind protocol (neither participant nor the investigator were aware whether the stimulation is real or sham).

Meta-analyses and reviews on acute tDCS effects on cognition performed so far provide mixed results about their capacity to modulate cognitive performance. While some studies report small to moderate beneficial effects (Dedoncker et al., 2016; Hill et al., 2016; Mancuso et al., 2016), others report no effects using tDCS within one single session in healthy young humans (Horvath et al., 2015) or when individuals perform working memory tasks (Medina and Cason, 2017). However, due to various experimental designs regarding electrode configurations, intensities, durations, electrode size and state-dependency, no general assumption on efficacy appears to be valid until more clarity is gathered (Jacobson et al., 2012; Tremblay et al., 2014). It is widely accepted within the motor domain that anodal stimulation of motor regions facilitates neural networks and that cathodal stimulation inhibits neural networks engaged in several motor tasks (for a review see Buch et al., 2017). This dichotomy is not yet well established for the stimulation of non-motor regions such as prefrontal areas. Consequently, some authors argue that tDCS-effects observed in the motor-domain cannot simply be transferred to the cognitive domain (Miniussi et al., 2008; Jacobson et al., 2012). Nonetheless, combined neurophysiological and behavioral studies present evidence for altered neural excitability (Nitsche and Paulus, 2000; Nitsche et al., 2005) and comparable polarity-specific effects in the PFC (review in Wörsching et al., 2016). However, one major caveat of tDCS is the high inter-subject variability suggesting that tDCS effects are dependent of individual factors, such as the instantaneous state of the brain (Antal et al., 2008; Dutta, 2015; Li et al., 2015) or genetic variations (Plewnia et al., 2013).

In terms of executive function research, few studies (review in Strobach and Antonenko, 2017) have investigated shifting ability using 10–30 min of tDCS, while only two have shown specific task-shifting effects (Leite et al., 2011, 2013). Leite et al. (2011), using two different shifting tasks, observed that stimulating the DLPFC either with an anodal or cathodal electrode configuration modulated the response speed of the shifting ability. The active electrode was placed over F3 (F3 refers to the electrode position according to the 10-20 international system for electrodes positioning) and the return electrode over the contralateral supraorbital area. Anodal stimulation improved the task performance, while cathodal stimulation decreased the task performance. Using cross-hemispheric tDCS stimulation (i.e., either left PFC anodal and right PFC cathodal electrode positions or vice versa) on two task-switching tests in a subsequent study, Leite et al. (2013) concluded that such effects were critically dependent on the laterality and the task. This was deduced because performance improvements in accuracy and speed of processing were reversed based on whether the right or the left PFC was stimulated, and which of the two task-shifting tests were used (Leite et al., 2013).

More evidence exists for the ability to update information in the working memory. Several studies have shown that this ability can be positively modulated by tDCS applied to the left DLPFC with anodal tDCS lasting 10–30 min (Fregni et al., 2005; Ohn et al., 2008; Andrews et al., 2011; Teo et al., 2011; Zaehle et al., 2011b; Hoy et al., 2013; Meiron and Lavidor, 2013). Those studies indicate that especially the left DLPFC is critically for working memory tasks. More specifically, Ohn et al. (2008) found significantly increased accuracy scores for a verbal 3-back task during and even 30 min after stimulation. Also, in the Fregni et al. (2005) study, positive effects on working memory performance were observed after only 10 min of tDCS. Comparable positive effects in clinical trials for one stimulation session were also found in patients with Parkinson’s disease (Boggio et al., 2006; Fregni et al., 2006c). Notably, in the Boggio et al.’s (2006) study only 2 mA but not 1 mA, yielded any performance improvements, indicating intensity-dependent effects. Intensity-dependent effects on working memory capacity (n-back task) were also observed in healthy participants in the Teo et al. (2011) study. Thus, tDCS current intensity is a critical factor that should be considered in health and disease tDCS interventions. Another interesting finding reported by Gill et al. (2015) showed that higher demands on the cognitive systems during tDCS had a significant effect on post-stimulation performance, indicating that the task and the timing of stimulation is an additional critical factor to consider. Not only left DLPFC, but also right DLPFC could be a potential target area for improving working memory performance as anodal tDCS over right DLPFC improved performance within a spatial working memory task, and particularly in the more complex task components (Wu et al., 2014).

There is also some evidence that tDCS can modulate the inhibition ability when the right PFC hemisphere is stimulated by anodal tDCS lasting 10–20 min as indicated by improved response inhibition (i.e., reaction times) in Go/No-Go and Stop-Task paradigms (Jacobson et al., 2012; Kwon et al., 2013; Hogeveen et al., 2016). In contrast, when using the Stroop task, increased performance has been identified by right DLPFC anodal tDCS and by left DLPFC anodal tDCS (Jeon and Han, 2012). Both anodal tDCS-initiated (right and left hemisphere) improvements were observable as long as 2 weeks after the stimulation session. Another study also using the Stroop task and a bilateral left anodal/ right cathodal tDCS electrode configuration, observed a positive effect in the response speed (Loftus et al., 2015). Other studies by Zmigrod et al. (2016), using the Eriksen flanker and Simon test (inhibition function), showed that cathodal tDCS over the right DLPFC influenced performance of the flanker test, but not of the Simon test (Zmigrod et al., 2016). An additional study also showed that only cathodal tDCS over the right DLPFC, but not anodal tDCS, influences the control of impulsiveness (Beeli et al., 2008). While the latter two studies showed selective tDCS effects (i.e., only cathodal effects), Hsu et al. (2011) reported that both anodal and cathodal tDCS over the superior medial frontal cortex either improved or deteriorated inhibitory control ability compared to sham stimulation. There are also some studies that explored prefrontal tDCS effects on more complex EFs measures such as the TOL, a test that taps into multiple EFs abilities. (Welsh et al., 1999; Miyake et al., 2000; Snyder et al., 2015). Dockery et al. (2009) found that 15 min of both anodal and cathodal tDCS over the left DLPFC improved planning performance measured by the TOL. Interestingly they observed that these effects were phase dependent, i.e., cathodal tDCS improved performance in an early learning phase while anodal tDCS was effective only in a later phase, suggesting training-phase-specific effects (Dockery et al., 2009). Comparable TOL improvements (initial thinking time) were found in a prefrontal bilateral tDCS protocol with anodal left DLPFC (cathode placed on the right DLPFC) improved TOL performance but cathodal left DLPFC tDCS had no effects (Heinze et al., 2014). Although so far only few studies exist, taken the reviewed single studies and reviews together there is some evidence that tDCS can modulate all three EFs even within one single application. However, due to different stimulation protocols (intensity, duration and timing), other non-significant effects and one contrasting meta-analysis (Horvath et al., 2015; Strobach et al., 2016), much more research is necessary to be certain how EFs are modulated by tDCS.

## Neurophysiological and Neurochemical Effects of Acute Exercise

Early animal studies on the exercise-cognition interaction proposed that increased arousal due to aerobic whole-body exercise increases brain concentration of neurotransmitters such as noradrenaline. This, in turn, activates the reticular formation, a heterogeneous and not well-defined structure of the nervous system that contains various nuclei associated with neurotransmitter releases. Due to this function (for a historical overview see McMorris, 2016b) in the noradrenergic, serotonergic and the dopaminergic pathways, the reticular formation is integral to the arousal activation system (review in Meeusen and De Meirleir, 1995). Currently, it is thought that only one session of exercise induces the synthesis and release of diverse neurochemical substances such as noradrenaline, adrenaline, dopamine, brain-derived neurotrophic factor (BDNF), insulin-like growth factor 1 (IGF-1), lactate, vascular endothelial growth factors (VEGF) and hypothalamic–pituitary–adrenal axis (HPA) hormones (e.g., cortisol) and, as shown in animal studies, they can potentially pass either directly or indirectly through the brain-blood-barrier to modify the arousal level and neuroplastic mechanisms (McMorris and Hale, 2015; McMorris et al., 2016; Basso and Suzuki, 2017). This modification, which is emphasized and detailed in the catecholamine hypothesis first proposed by Cooper (1973) and further developed by works of McMorris (Cooper, 1973; McMorris et al., 2008; McMorris, 2016a), leads to either the optimal or suboptimal preparation of a person for action and aids in neurogenesis and neuroplasticity.

The catecholamine hypothesis (McMorris et al., 2008) provides a rational explanation for the discussed meta-analytical findings of moderate positive effects sizes in cognitive performance (McMorris and Hale, 2012). Based on the findings of neurochemicals associated with acute bouts of exercise, it is thought that elevated concentrations of the neurotransmitter’s dopamine and norepinephrine observed during and following moderate-intensity exercise, should theoretically facilitate cognition. However, observations of catecholamine releases (and thus reticular system) in response to low-intensity AE indicate that processing speed in various cognitive tasks is reduced due to less activation in the relevant brain areas. In contrast, high-intensity exercise induces more massive catecholamine releases during and following the exercise session and introduces neural noise, ultimately leading to decreased cognitive performance. However, such an inverted-U relationship has never been unequivocally observed, and recent considerations on this hypothesis are only conceivable by adding data from animal studies (McMorris et al., 2008; McMorris, 2016a).

More specifically, moderate increase of catecholamines due to moderate exercise intensity mostly performed between 20 min and 30 min were optimal for working memory improvements. In contrast, longer lasting moderate exercise provoked interaction between HPA hormones and catecholamine, leading to the inhibition of cognitive performance (McMorris et al., 2016). Furthermore, high-intensity AE sessions induced activation of β-adrenoreceptors in the hippocampus and lead to increased memory performance and elevated BDNF concentrations (Neeper et al., 1995; Piepmeier and Etnier, 2015; Szuhany et al., 2015), agreeing with the observation that a short period of high-intensity exercise increased BDNF more than a long-lasting moderate exercise intensity session (Winter et al., 2007). Single studies are confirmed by meta-analytic evidence with moderate effect sizes showing BDNF releases after only one-session exercise (Szuhany et al., 2015). In addition, suggesting against an exercise-intensity dependence of BDNF release (review in Piepmeier and Etnier, 2015), low-intensity exercise elevated BDNF in the same amount as in a high-intensity condition. In contrast, Ferris et al. (2007) confirmed the exercise intensity dependence of BDNF release after 30-min cycle exercise, but could not find any correlation of BDNF changes with EFs, such as inhibition ability measured by a Stroop test (Ferris et al., 2007). Moreover, no intensity effect was observed since the Stroop task performance improved pretest- to posttest at the same extent, regardless of exercise intensity. In a recent comprehensive review of acute exercise effects on neurophysiological parameters, it was reported that there is consistent observation that high exercise intensity around the anaerobic threshold induces approximately 15% increases in BDNF which last up to 20 min after exercise. However, one limitation of studies on intensity-dependent BDNF effects in humans is the risk that peripherally measured BDNF may not sufficiently reflect cortical BDNF level (Basso and Suzuki, 2017).

There are also many electroencephalography (EEG) studies investigating tasks that evoke EEG responses during, immediately after, or with a time delay after AE. They are mostly quantified using event-related potentials (ERPs) P300, contingent negative variation (CNV), error-related negativity (ERN) and error positivity (EP). P300 is a prominent marker that can be seen as a positive deflection in the EEG amplitude about 300 ms after a stimulus and P300 is an index of attentional resources devoted to task completion (Polich, 2007). Thereby, difficult and complex tasks reduce P300 and increase latency. Kamijo et al. (2004a), as an example, observed decreased P300 amplitude after a high-intensity cycling exercise in a Go/no-go task and increased P300 amplitude after moderate intensity exercise (Kamijo et al., 2004a). Based on this pattern, they suggested that reduced attentional resources were available after high-intensity exercise and increased resources after moderate exercise. In another study by Kamijo et al. using the CNV as a marker of attention and arousal, they found further evidence for intensity dependent effects of exercise on central neurocognitive markers (Kamijo et al., 2004b).

By using additional markers such as the ERN and EP (markers observable in evaluation of conflict during instances of erroneous), those ERPs clearly demonstrate that the exercise-cognition relationship is reflected in task-evoked brain activity (Yanagisawa et al., 2010; Chang, 2016), with further evidence for intensity-dependent effects (Olson et al., 2016). In contrast, early stimulus-locked components of the EEG such as N1 and P2 (reflecting early processing steps that are not directly related to EFs) are not affected by exercise, while P300 and CNV reflect exercise-associated behavioral changes. A review by Chang (2016) concluded that a moderate exercise protocol lasting between 18 min and 30 min exerts beneficial effects on cognition and neurocognitive markers (Hillman et al., 2003, 2009; Scudder et al., 2012; Drollette et al., 2014).

Moreover, oscillatory EEG (i.e., the synchronized activity patterns of neurons in a functional neural network) activity recorded during and after exercise indicated increases in the alpha band (8–12 Hz oscillation indicating a marker of arousal); however, this was not different from the increase measured for other frequency bands (Crabbe and Dishman, 2004). Subsequent research observed frontal alpha asymmetry (FAA) changes. FAA is a marker of different activity in the left and right PFC hemispheres, and this study indicated higher relative left prefrontal activity elicited by moderate and strenuous exercise intensity. This pattern was measurable up to 38 min post-exercise and associated with post-exercise mood modification (Woo et al., 2009; Hall et al., 2010; Hicks et al., 2017). Gutmann et al. (2015), for example, showed that the resting state individual alpha peak frequency (iAPF), which is a marker of arousal and attention and is associated with the speed of information processing, was increased after an exhaustive exercise compared to steady state moderate exercise intensity. This finding suggests there is a mechanism leading to an optimal brain state for cognitive performance (Gutmann et al., 2015). Furthermore, resting-state functional magnetic resonance imaging (fMRI) analysis proposes that AE increases the integration of attention and executive control networks indicative of functional network connections that are particularly sensitive to moderate-intensity AE (Weng et al., 2017). A review on acute exercise effects including animal and human studies summarized that a single bout of exercise increases hippocampal theta activity and other frequency bands across the entire cortex, positively modulates the P300 and indicates that cognitive enhancement is accompanied by increase in cerebral blood flow (Basso and Suzuki, 2017).

However, no clear association exists between brain oxygenation, cerebral blood flow, and cognitive performance during exercise, although alterations of these parameters have repeatedly been reported (review in Ando, 2016). This conclusion contrasts with the influential reticular-activating hypofrontality model (Dietrich, 2003; Dietrich and Audiffren, 2011). This complex theory principally suggests that the human brain has a limited information-processing capacity that eventually leads to decreased cognitive performance with rising exercise intensity. According to this model, moderate exercise intensity leads, through activation of the reticular system, to increased arousal, and in turn, to increased cognitive performance, but this effect only applies in tasks that are well-learned. As soon as the intensity is high, the model proposes that motor areas of the brain must be higher activated due to resource allocations for motor task completion. If exercise intensity level is to be maintained, this can only occur at the expense of the PFC areas, which are downregulated. In turn, this PFC deactivation results in poorer cognitive performance especially in tasks that rely on PFC structures such as EFs. However, this well-defined model, while it has some striking empirical support (for an overview see Dietrich and Audiffren, 2011), only accounts for any online (effects observed during AE) exercise-cognition interactions (i.e., limited explanatory power for any offline effects), and there are other empirical findings, which disagree with this model (e.g., Tempest et al., 2017).

## Neurophysiological and Neurochemical Effects of tDCS

In tDCS, the transcranially induced, weak, direct current flows from the anode to the cathode, passing the neural tissue, and is thought to exert transient alterations in the neural processes of the stimulated brain region. Although the exact physiological mechanisms of tDCS are still being studied, it is presumed that the primary mechanism of action derives from a polarity-specific subthreshold polarization of the resting membrane potential of neurons (Nitsche and Paulus, 2000, 2001). Recently, it was indicated by intracranial recordings in primates and epilepsy patients that 1–2 mA of anodal tDCS slightly elevated the resting membrane potential by approximately 0.2–0.5 mV (Opitz et al., 2016). In turn, such a transiently increased membrane excitability is suggested to boost the likelihood of action potentials (APs), resulting in a higher spontaneous firing rate of neurons. In contrast, cathodal tDCS exert the opposing effect of a transient hyperpolarization, thereby more likely diminishing cortical excitability and the spontaneous firing rate of neurons (Creutzfeldt et al., 1962; Bindman et al., 1964; Nitsche and Paulus, 2000; Romero Lauro et al., 2014).

Consequently, anodal tDCS is typically associated with increased cortical excitability, while cathodal tDCS is associated with decreased cortical excitability. The polarity-specific effects of tDCS on resting membrane potentials originate from tDCS-induced changes in the conductivity of voltage-dependent ion channels. Anodal tDCS is assumed to increase cortical excitability due to modulations of N-Methyl-D-Aspartate-receptor-mediated (NMDA) Ca^2+^-channels, causing alterations in Ca^2+^ influx (Nitsche et al., 2003a). However, polarity-specific alterations of cortical excitability are predominantly demonstrated in motor regions of the brain (e.g., M1), such as by higher amplitudes of TMS-induced motor-evoked potentials (MEPs) (Nitsche and Paulus, 2000, 2001). Excitability changes were also observed in non-motor regions by means of sensory-evoked potentials (SEPs) in the somatosensory cortex (Dieckhöfer et al., 2006), auditory-evoked potentials (AEPs) in the auditory cortex (Zaehle et al., 2011a), and visually-evoked potentials (VEPs) in the visual cortex (Antal et al., 2004).

Short durations of tDCS application can elicit effects that last a few seconds, whereas longer-lasting tDCS application can prolong the after effects on membrane excitability and cortical activity. Exemplarily, it was demonstrated that 13 min of anodal and 9 min of cathodal tDCS can initiate long-lasting after-effects for 30 to 120 min (Nitsche and Paulus, 2001; Nitsche et al., 2003b; Kidgell et al., 2013) and can last up to 24 h when tDCS was repeatedly applied throughout 1 day (Bastani and Jaberzadeh, 2014). Longer-lasting after-effects of tDCS are rendered to provoke neuroplastic processes, as indicated by tDCS-related modulations of several neuroplasticity markers, such as glutamate (Nitsche et al., 2003a), gamma-aminobutyric acid (GABA) (Nitsche et al., 2004), dopamine (Nitsche et al., 2006; Monte-Silva et al., 2009), serotonin (Nitsche et al., 2009), acetylcholine (Kuo et al., 2007) and BDNF (Fritsch et al., 2010). In the corresponding primary mechanism of action, anodal tDCS causes a reduced concentration of GABA with a concurrent increase in glutamate concentration, while cathodal tDCS brings about the opposing effect (Stagg et al., 2009; Stagg and Nitsche, 2011). Furthermore, the reduced GABA concentration and the concurring reduced GABA-gated intracortical inhibition (ICI) provoked by anodal tDCS leads to a facilitative effect on glutamate-driven neuroplasticity (Stagg et al., 2009; Kim et al., 2014). Therefore, tDCS-related neuroplasticity appears mainly as a glutamatergic process comprising NMDAs, and is based on their numbers upon glutamatergic synapses causing increased synaptic strength and responsiveness to glutamate (Gillick and Zirpel, 2012), and NMDA-mediated influx of Ca^2+^ (Liebetanz et al., 2002; Nitsche et al., 2003a). Fluctuations of Ca^2+^ are further associated with both long-term potentiation (LTP) and long-term depression (LTD) (Bennett, 2000), synaptic processes, which are contingent upon BDNF (Fritsch et al., 2010).

In addition, fMRI, EEG, and functional near-infrared spectroscopy (fNIRS) approaches reported local functional synchronization due to tDCS (Polanía et al., 2011, 2012; Kunze et al., 2016) and altered brain activity in nearby and functionally connected brain areas (Baudewig et al., 2001). fMRI studies observed extended and large-scale network changes following one session of cathodal stimulation (Ardolino et al., 2005; Lang et al., 2005). fNIRS approaches reported hemodynamic changes after prefrontal anodal tDCS (Merzagora et al., 2010) and EEG recordings found that the oscillatory activity of the brain adapts its frequency in response to tDCS (Keeser et al., 2011; Zaehle et al., 2011b; Jacobson et al., 2012). In particular, anodal tDCS applied over the left DLPFC increased the cortical perfusion in the stimulated area, while a strong decrease was observed after the stimulation (Stagg et al., 2013). Moreover, prefrontal tDCS reduces low-frequency EEG oscillations and increases event-related activity (for an overview see Wörsching et al., 2016). Also, surface brain area activity changes and alterations in neural processing in deeper prefrontal structures have been described (Keeser et al., 2011). Studies on resting-network activities consistently report tDCS induced influences on regional activity on functional connectivity across brain regions interhemispheric and intrahemispheric (Turi et al., 2012; Hartwigsen and Siebner, 2013; Hartwigsen et al., 2015; Bergmann et al., 2016; Kunze et al., 2016).

Further neurophysiological measures during EF execution (inhibition tasks, a combined Go/no-go and Stop signal task) using a combined tDCS-EEG approach observed that an anodal bilateral tDCS (right hemispheric anodal/left hemispheric cathodal) of the right inferior frontal cortex (IRF) modulated the P300 ERP component, while no clear effects were observed on a behavioral level (Cunillera et al., 2016). This study was one of the first to report EEG data during the stimulation as the EEG is usually compromised by the tDCS electrical field. Although P300 was affected by tDCS, the authors concluded that bilateral tDCS of the right IRF is not the best option to modulate response inhibition. Another study targeted the medial frontal cortex with cathodal tDCS and induced EP modifications of the EEG (Bellaïche et al., 2013). Since the EP reflects error monitoring, the authors concluded that targeting those brain areas involved in error monitoring with a neuromodulatory technique such as tDCS would be valuable for therapy. This conclusion was based on the error monitoring ability in patients suffering neuropsychiatric conditions being frequently impaired. Keeser et al. (2011) observed modulations of the P300 ERP component of the EEG, together with behavioral improvements in an updating ability task (N-back) after anodal tDCS (cathode placed over the left supraorbital cortex) of the left DLPFC (Keeser et al., 2011). Further source analysis using EEG based standardized low resolution tomography (sLORETA) revealed enhanced para-hippocampal gyrus activity, suggesting that tDCS alters the regional surface neural activity and reaches deeper PFC structures.

Based on the reviewed studies targeting the PFC, there is converging evidence that prefrontal tDCS impacts resting state activity, task-evoked activity, brain oscillations, brain perfusion and oxygenation, bioenergetics, functional connectivity, event-related spectral perturbations (ERSPs) and ERPs. Consequently, tDCS of the PFC initiates comparable synaptic-plasticity and associated neuroplastic markers known from motor cortex studies (see Wörsching et al., 2016). However, direct measures of excitability changes using TMS, which induces a direct measure of cortical excitability (i.e., MEPs), are not available for prefrontal structures.

## Interactions and Synergies Between Acute tDCS and Acute Periods of Aerobic Exercise

Converging evidence exist indicating that both tDCS and AE involve promising mechanistic pathways that initiate changes in cortical excitability and neuroplasticity that can improve cognitive functions even in the short-term. Therefore, it is conceivable that a combination of both techniques could act in a synergistic fashion to improve cognitive performance beyond the level known for each technique alone. The idea of combining multiple techniques is far from being new, as several recent studies and reports have shown complementary effects when tDCS and exercise are combined in an interventional approach (Okano et al., 2015; Angius et al., 2017; Edwards et al., 2017; Ward et al., 2017). There are also several other multimodal interventions using AE without tDCS, aimed to improve cognitive functions. For example, a direct combination of meditation with AE mitigated symptoms of major depressive disorder, provoked enhanced neurocognitive markers (P300) and indicated increased synchronous brain activity during a cognitive control task (Alderman et al., 2016). Thus, similar to meditation, tDCS may synergistically modulate neural networks in combination, as this technique has been shown to mitigate depressive symptoms, increasing cognitive functions and modifying neural activity (Fregni et al., 2006b; Schuch et al., 2016). Other more direct interventions with the purpose of improving cognitive functions have combined cognitive training with AE training, either in a direct combination (i.e., cognitive training during exercising) or in an indirect fashion (i.e., sequentially). In a meta-analysis of studies with older aged participants, mixed results compared to single interventions appeared; however, this result could have been due to the heterogeneity of training protocols (Zhu et al., 2016). In particular, studies applying a sequential approach have either revealed positive effects on EFs (Rahe et al., 2015; Lai et al., 2017) or no superior effects on EFs compared to single-modality training (Shatil, 2013). Importantly, the latter study applied AE training and cognitive training on different days, while the other two studies combined cognitive and AE training in one session by having cognitive training followed directly by AE. Thus, one bout of AE and its acute effects could initiate an ideal environment for increases in a subsequent training of EF or other cognitive functions and might thus serve as a brain primer for subsequent tDCS intervention.

In line with the presented argumentation that multimodal interventions provide a possibility for initiating neuroplastic processes and superior performance gains compared to single-modality interventions based on the literature reviewed thus far, AE might be capable of positively interacting with the tDCS induction of synaptic plasticity. It could well be, although not tested so far, that combining these techniques may enhance synaptic processing and network activity that are associated with EFs. For example, following the catecholamine hypothesis, a session of moderate AE intensity elicits an ideal amount of catecholamine release for cognitive enhancement. This enhancing pathway is potentially mediated through catecholamine, because pharmacological studies have shown that central EF tasks require the activation of the noradrenergic and dopaminergic pathways (Luciana et al., 1998; Berridge et al., 2006; Chamberlain et al., 2006). The enhancement of EFs by anodal tDCS during stimulation (i.e., online), however, is thought to be based primarily on changes in resting membrane potential (Stagg and Nitsche, 2011). Those divergent mechanisms, capable of initiating cognitive enhancement on its own, might be one possibility when combined in one session for synergistic effects on EF performance. While tDCS may modulate the frontal neural networks in a more specific and focused manner when stimulating the PFC structures such as the DLPFC [e.g., even more focally using smaller electrodes, i.e., high-definition tDCS systems (Datta et al., 2009; DaSilva et al., 2011; Villamar et al., 2013)], the AE may activate broader networks through reticular arousal activations pathways and neural oscillatory modifications (Hall et al., 2007; Woo et al., 2009; Gutmann et al., 2015; McMorris and Hale, 2015; McMorris, 2016a,b; Basso and Suzuki, 2017; Hicks et al., 2017). The involvement of the two converging pathways may then, as an example, enhance EF inhibition. In particular, brain areas involved in inhibition, such as the ACC (van Veen et al., 2001; Mansouri et al., 2009; Kühn et al., 2016; Weng et al., 2017), are not directly accessible by tDCS, but they can be reached by arousal activation through physical exercise (Critchley, 2004). This, in turn, might be supportive in a synergistic fashion for the executive function ability inhibition and possibly other EFs.

Alternatively, AE performed directly prior to an anodal tDCS session of the PFC, in which EFs are trained or tested may act complimentary for the induction of neuroplasticity based on BDNF expression. The increase in BDNF might create an optimal environment for longer lasting tDCS induced LTP initiation. As BDNF factors are necessary for successful and efficient LTP induction (Cotman and Berchtold, 2002), these pathways (AE initiated BDNF and tDCS initiated LTP) might converge into optimal learning processes and synaptic plasticity. In particular, animal studies at which rodents performed exercise sessions have indicated that BDNF levels in the hippocampus are related to enhanced learning and memory processes (Vaynman et al., 2004), and, as reviewed, BDNF expression in humans due to exercise has been repeatedly observed (Winter et al., 2007; Szuhany et al., 2015). Cortical concentrations of BDNF are reduced concomitant with disturbed LTP mechanisms in several diseases, such as Alzheimer’s disease, Parkinson’s disease, depression, anorexia, and many other diseases (Mariga et al., 2017). Thus, AE in combination with tDCS might act for disease prevention or to enhance cognition and cognitive training regimens and restore maladaptive neural functions in such diseases. However, in specific cases, and based on the targeted system, the AE intensity plays an additional critical role since high-intensity exercise has several positive effects such as a superior elevation of BDNF compared to moderate intensity. Exercise-induced BDNF increase in general (Piepmeier and Etnier, 2015; Szuhany et al., 2015) is higher due to heavy exercise compared to a longer-lasting moderate exercise session (Winter et al., 2007). Thus, the exercise intensity may have a critical role for initiating/restoring optimal neuroplasticity, which is mediated by BDNF, such as in major depression (Brunoni et al., 2008), a psychiatric state which can be influenced by tDCS and AE (Fregni et al., 2006b; Kuo et al., 2014; Schuch et al., 2016).

Due to heterogeneous findings on cognitive abilities during exercise, it is only possible to speculate about how this cognitive-exercise interaction could be additionally modulated with parallel anodal or cathodal tDCS. Although the tDCS-induced electrical field might be affected during whole body movements through movement artifacts and head transpiration, behavioral modifications due to stimulation during exercise have already been observed repeatedly (Angius et al., 2017). Several recent works provide first evidence that single bouts of tDCS can improve exercise performance (for an overview, see Angius et al., 2017; Edwards et al., 2017). In most of these studies (9 out of 12), anodal tDCS was applied to the left or right M1 and the cathode was mostly placed at an extra-cephalic position (e.g., shoulder) or a contralateral site, such as the contralateral forehead. In two studies, the anode was placed over the temporal area (T3) and in another study in a central position (Cz). However, with the exception of two studies, anodal tDCS was applied prior to the exercise protocol and lasted for 10–20 min. In sum, the studies indicated mixed results but point toward a tendency to improve exercise performance following tDCS application. Primarily, it is thought that tDCS reduces and delays supra-spinal fatigue accompanied by a reduced subjective perceived exertion (Williams et al., 2013; Okano et al., 2015; Angius et al., 2016). Therefore, one additive effect of tDCS and AE might emerge due to the fact that people can endure higher exercise intensities, especially in aged or clinical populations, when tDCS is applied to the motor cortex for example prior to an AE session. As outlined, heavy AE seems to be more beneficial in terms of BDNF releases compared to moderate AE (Basso and Suzuki, 2017) which, in turn, may have a stronger effect on cognitive performance.

However, to the best of our knowledge, no study has investigated a possible interaction between exercise, tDCS, and cognition. Therefore, only indirect conclusions for the cognitive domain can be derived from combined tDCS-AE studies within the motor- and sports-related domain. To the best of our knowledge, so far, there is only one published RCT trial that implemented in a multimodal study design a group that received tDCS and AE treatments (Ward et al., 2017). In this comprehensive 4-month study, cognitive training success (computer-based EF and working memory training) was compared between five different groups, whereas one of those groups received tDCS over DLPFC (HD-tDCS: two small anodes were placed on left and right PFC and two return electrodes at occipital areas) during cognitive training and a physical exercise training. The rationale is that previous studies have shown that cognitive training success accompanied by tDCS or by AE training outperformed cognitive training in isolation (Ward et al., 2017). When they are combined in one group, converging mechanisms, as reviewed in this work (LTP, neural excitability, network modifications, and synaptic plasticity) and addressed by Ward et al. (2017), should ultimately be expressed in enhanced cognitive learning success compared to unimodal interventions (e.g., only cognitive training). Indeed, they found that the combined tDCS-exercise group outperformed other unimodal and multimodal groups in cognitive performance, suggesting synergetic effects on the cognitive system. Unfortunately, there is no specific information about the time delay between the exercise session and the tDCS-cognitive training session. Moreover, exercise sessions also included resistance training and non-standardized AE training protocols, making it difficult to clearly conclude whether the higher cognitive training effects were due to the combinatory effects evoked by each single technique alone, or whether there were any direct and acute interactions between exercise and subsequent tDCS effects (e.g., exercise that serves as a brain primer; more details regarding this aspect are in the next paragraphs).

One key parameter for the interaction between tDCS and AE seems to be the timing of the AE and tDCS sessions, i.e., whether tDCS is applied prior, during, or after AE. Based on tDCS studies stimulating the motor areas, the timing of tDCS relative to a given task seems to be a crucial factor with a strong impact on study outcomes (Stagg and Nitsche, 2011). In experiments where tDCS is applied during task performance (online tDCS), the specific neural network involved to perform the task is mainly stimulated and the tDCS-induced neuroplastic changes primarily occur within the task-related neuronal circuits (Huang et al., 2017). However, studies indicated behavioral improvements following online tDCS and after tDCS administration in the absence of task performance (offline tDCS). As reviewed, the timing of AE with regards to cognitive test administration is important, but the timing interacts with exercise intensity especially during AE (i.e., online). In addition, both the online and offline cognitive testing within AE seem to be an option to enhance behavioral output, as AE effects may serve as an ideal primer to initiate an optimal environment to improve brain functions in both cases. No explicit knowledge is available for the best timing of tDCS and AE. However, if an intervention wants to exert the online-effects or any after-effects of one technique directly on another technique, then the timing appears to be crucial, considering the observation of tDCS after-effects of no longer than 120 min (Nitsche and Paulus, 2001; Nitsche et al., 2003b; Kidgell et al., 2013).

Further hints for the optimal timing come from priming studies (Parkin et al., 2015), since a specific kind of priming or “pre-conditioning” (a task or intervention applied before a second “conditioning” task or intervention) is thought to initiate “metaplastic” processes by using a short priming (or pre-conditioning) period of tDCS stimulation before the actual stimulation (TMS or tDCS) is applied. There is emerging evidence from motor, cognitive, and vision studies that metaplasticity provokes more robust results and even boosts effects of conventional protocols (Hurley and Machado, 2017). The term metaplasticity refers to the Bienenstock et al. (1982) model (BCM) of synaptic modification. The BCM implies that a preceding inhibition of cortical activity decreases the threshold of a subsequent excitation and vice versa for a preceding elevation of cortical excitation (i.e., increased threshold for excitation) (Bienenstock et al., 1982). Consequently, the plasticity of the state of a neuron depends on earlier states induced by separate prior events (Abraham, 2008; Hulme et al., 2013). It is thought that such an experience-dependent neural plasticity mechanism requires the existence of a state-dependent fast-reacting system that can dynamically adapt in response to preceding synaptic activity to retain network stability and permit enduring LTP or LTD-like mechanisms (Abraham, 2008; Feldman, 2009).

Thus, the modification or state of a neural network prior to an intervention can have an impact on a subsequent intervention where the same network is targeted (within the timeframe of after-effects). This mechanism was tested by Carvalho et al. (2015) using two tDCS sessions with a time delay and different polarities in a working memory task (N-back) (Carvalho et al., 2015). Notably, anodal tDCS over the DLPFC as a pre-conditioning period decreased working memory performance during subsequent anodal tDCS of DLPFC (conditioning stimulation). This decrease occurred when a time delay was placed between the two stimulations; thus, metaplastic processes may have changed the direction of polarity. In contrast, using cathodal tDCS as a primer 10 min before a second cathodal tDCS conditioning stimulation enhanced working memory performance, suggesting a metaplastic mediated compensation with an upregulation process due to the prior inhibition period induced by cathodal tDCS. Thus, the effects reversed, agreeing with the BCM model for retaining network stability and a phenomenon called the “rebound effect” (Creutzfeldt et al., 1962). Consequently, due to the aforementioned AE acute effects, a short bout of high-intensity AE, as an example, could be an additional tool and taken as a primer (pre-conditioner) for subsequent anodal or cathodal tDCS. Considering that one bout of AE increases excitability of the brain and increases the aforementioned BDNF levels, AE might synergistically interact with LTP-like mechanisms induced by metaplasticity. Therefore, either AE or tDCS could be considered a versatile tool to initiate a specific desirable state of the brain. Thereby, AE—with its possibility to modify the excitability of the brain could be used as a brain primer, and tDCS could be used as a therapeutic tool to synergistically target specific brain areas more specifically. Alternatively, AE could be applied after a tDCS phase to facilitate consolidation in the hours after tDCS.

A further aspect to consider may be that meta-analyses provide evidence that moderate exercise intensity is most beneficial for cognitive functions, but affects only the speed of processing and not accuracy (McMorris et al., 2011; McMorris and Hale, 2012). Increases in catecholamine with working memory performance improvements were observable at between 20 min and 30 min of moderate exercise intensity, while longer lasting moderate AE resulted in cognitive inhibition (McMorris et al., 2016). For tDCS, studies show that offline stimulation improves accuracy in working memory tasks and helps neuropsychiatric patients during online stimulation to a greater extent than tDCS is able to improve processing speed (Dedoncker et al., 2016; Hill et al., 2016). Thus, there might be a synergistic pathway for a combined short and moderate AE session and subsequent tDCS to improve both the accuracy and speed of working memory performance.

Another potential pathway for its combined effect is the inhibitory system, as recent evidence shows that increased tonus of the inhibitory system is essential for increasing cognitive performance due to its important regulatory activity of multiple competing systems. Interestingly, the lack of cognitive demand leads to a lack of inhibition, likely due to a compensatory mechanism (Capano et al., 2015). Exercise seems to have a dual effect with an initial increase in excitatory circuits activity followed by an increase in inhibitory circuits, and has been used in children to increase inhibitory control, especially in cases of attention deficit hyperactivity disorder (ADHD) (Chang et al., 2014). Anodal tDCS seems to lead to a similar effect. Although anodal tDCS does lead initially to an increase in spontaneous neuronal firing, it is followed by an increase in intra-cortical inhibition (Nitsche et al., 2005; Vignaud et al., 2018). Thus, combining both therapies may enhance these effects; however, in this case, the timing of both therapies needs to be planned, as the AE session may not be during the compensation phase and increase in the inhibitory tonus. There is currently a lack of data to test this hypothesis, and simultaneous application of both therapies seems the best option (Mendonca et al., 2016).

### Future Implications

**Figure 1:**
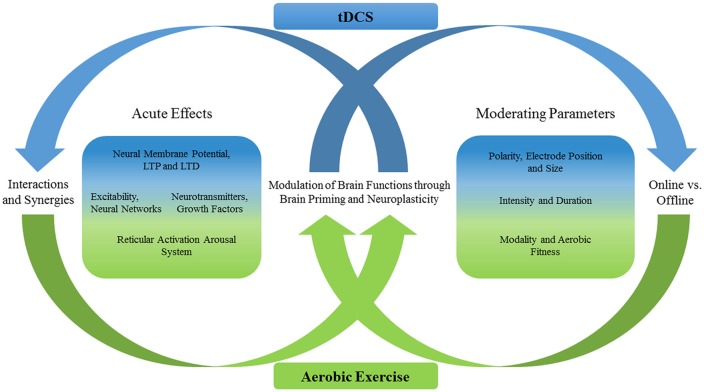
Acute effects of transcranial direct current stimulation (tDCS) and aerobic exercise (AE) and possible interactions. Shown is a schematic overview/ summary of the key concepts/effects reviewed in this article. The figure shows some acute effects and interacting parameters that could play a role when both techniques are applied in combination for supporting cognitive performance. The green color represents the AE effects and the blue color those of tDCS. The more a specific effect (or moderating parameter) is displayed in the middle of the two-colored boxes the more the effects are comparable between both techniques. This drawing does not focus on providing a comprehensive overview of all acute effects that have been observed for tDCS and AE (e.g., those for the motor, perceptual or affective domain). Instead, this figure includes those that might be supportive of the cognitive domain and those that should be critically evaluated in future multimodal approaches. LTP means long-term potentiation and LTD long-term depression.

The outcome of a direct combination of tDCS and AE (i.e., tDCS occurs either during or in a sequential fashion with AE) on a molecular and system level has not yet been investigated. However, the outcome of both techniques is modulated by many parameters, and all of which may be of relevance when both techniques are directly combined (see Figure 1 for an overview). Following our working hypothesis that moderating parameters need to be considered, the timing of cognitive test administration (or the training of EF) and the AE intensity are important factors to be investigated in the future with regards to whether tDCS and AE are sequentially or directly combined.

One practical argumentation of combining both techniques come from an applied perspective as both techniques have remarkable similarities besides converging mechanisms and modulatory capacity of brain functions. For both techniques, 20 min of application has been found to be especially supportive in initializing high effects with enhancement of EFs and other cognitive abilities. As an example, tDCS of the DLPFC or moderate-intensity AE of about 20 min have shown to enhance the EF inhibition, in both online and offline situations (Kamijo et al., 2007; Davranche et al., 2009; Loftus et al., 2015; Strobach and Antonenko, 2017). Moreover, the techniques can be performed in a direct combination (i.e., stimulation during exercising) when mobile wireless tDCS hardware is used. Critical aspects such as movement artifacts (compared to EEG or fNIRS) do not have a major role, and tDCS and AE are relatively easy to apply, cost-effective and without known severe side effects. Thus, they can be easily applied in almost all settings in real life, in health, in sport, peak performance, and in the treatment of disease (except in patients with contraindications) without any significant restrictions in body movement.

The various effects of both techniques on cognition and brain functions, their potential synergistic interactions, and their promising possibilities from an applied perspective suggest that further systematical investigations into the value of their combined use in health and disease is worthwhile. Because of the possibility of both tDCS and AE modulating brain functions and EFs, it would be desirable to investigate whether AE can prime the brain to foster an ideal brain state for optimized brain stimulation using tDCS. Thus, if a method wants to prime the brain or aims to initiate meta-plasticity processes by combined tDCS/AE, exercise intensity and duration (e.g., long moderate or short high intensity), timing (i.e., the delay between exercise and tDCS session), sequence (i.e., exercise before tDCS or after tDCS), and polarity (i.e., cathodal or anodal) of tDCS are critical factors to be considered. Regardless of metaplasticity, depending on the study objective or clinical objectives, whether downregulation of a given brain area (such as in depressive patients) or upregulation (such as in cognitive training for healthy people) is intended, should be carefully considered.

Especially with respect to the reticular activation hypofrontality theory (see section “Neurophysiological and Neurochemical Effects of Acute Exercise”), it might also be worth systematically modulating prefrontal cortical activity with tDCS or transcranial alternating stimulation (tACS) to actively upregulate or downregulate PFC activity during exercise. Using tDCS or tACS (through application of alternating current to brain areas, this technique aims to modulate brain oscillations and thus the functions that are associated with specific frequency bands) as a versatile tool and possibility for making causal inferences may help to confirm or reject the idea of rising hypofrontality with increasing exercise intensity (Dietrich and Audiffren, 2011). However, if there is a downregulation of the PFC by high AE intensity according to this hypofrontality model, a combined cathodal tDCS might synergistically support the down regulative capacity of AE on PFC structures. This could be experimentally investigated and actively used to optimize AE treatment protocols that have been shown to help depressive patients (Melo et al., 2016), possibly by the pathways defined in the hypofrontality model (Dietrich and Audiffren, 2011). In turn, anodal tDCS administered to the motor cortex during high AE intensity may release brain capacities which are available for cognitive functioning. The increased motor cortical activity required for intensive motor performance, might be supported by increasing motor cortical activity with anodal tDCS applied over the motor cortex. Hence, one may speculate that the higher cortical activity induced exogenously by anodal tDCS provokes lower needs for endogenous brain activity for motor performance, which in turn, release brain capacities (e.g., information-processing) and enhance cognitive performance during high AE.

Moreover, it might worth considering that modulations in brain oscillatory activity such as in the individual alpha band (e.g., FAA) can be provoked by exercise and tDCS and tACS (Zaehle et al., 2010, 2011b; Herrmann et al., 2013; Soekadar et al., 2016; Herrmann and Strüber, 2017; Kasten and Herrmann, 2017). Because several neurological and psychiatric conditions are associated with dysfunctional brain synchronization and maladjusted oscillatory communication (Stam et al., 2003; Schnitzler and Gross, 2005), a combined application of AE and tACS/tDCS could help research to modulate brain oscillations and study the associated behavior systematically.

Despite any potential and promising possibilities of a combined tDCS-AE use, however, there are several important aspects to consider. Although there is a multitude of studies indicating beneficial tDCS effects on cognitive function and possible synergistic pathways, there are also several studies suggesting no or even detrimental effects. Moreover, there are hints that the current state of the brain and dynamically changing brain physiology can have a significant impact on any tDCS-related effects, a factor which must be carefully considered and which might be especially susceptible by exercise and its moderating factors. As an example, one could assume that the effects elicited by a combined tDCS-exercise protocol on cognitive functioning may be contingent upon the individual fitness level, thus making it important to account for inter-subject variability. Moreover, the present review did not consider the different effects of tDCS and AE being the result of factors such as age (e.g., very young and old people), gender or specific neurological and psychiatric states, all of which might be differently affected by the two techniques and specifically by the moderating parameters reviewed here. Lastly, even if there are any potential synergistic combined tDCS-AE effects from one application, it must also be examined whether this has an impact on any longer lasting (i.e., repeated application of a combined AE-tDCS) interventional approaches, where additional factors must also be considered.

## Conclusions

AE and tDCS can have remarkably similar effects on EFs and other cognitive domains and support rapid initiating of neuroplasticity in the human brain within a short timescale. This similarity offers multiple beneficial opportunities within clinical research, such as treatment of psychiatric diseases or neuro-rehabilitation and also in non-clinical settings, such as sport. Compared to other multimodal approaches, such as combined AE-TMS studies or treatments combined with pharmaceutics, tDCS-AE has the advantages of its easy-to-apply and time and cost-effective applications without any yet known severe side effects. The acute effects of both techniques on the neurophysiological, neurochemical, and behavioral level, such as the enhancement of cognitive skills, modifications of neural activity, and catecholamine, have striking similarities and provide synergistic mechanistic pathways that might improve brain functions and neuroplasticity in health and disease when both techniques are applied in direct combination. While AE might provoke more large-scale changes across the entire brain, serving as a brain primer to provoke a given desired brain state, tDCS may then: (a) be used to more focally and specifically modulate brain activity and behavior; and (b) lead to more robust and higher effects possibly due to lower intersubjective variability and potential metaplastic effects.

Due to various interacting and dynamically changing parameters, caution must be given to those moderating parameters that could significantly impact the interaction between both techniques, possibly leading to the abolishment of any effect (see Singh et al., 2016). As outlined, moderating parameters of AE and tDCS, such as exercise and electric intensity, tDCS polarity, exercise and tDCS duration, and timing of tDCS, provoke modifications on several levels of the human organism, even with only one application. Both AE and tDCS are capable of modulating cognitive functions, brain activity, and excitation in terms of brain oscillations, hemodynamic activity, NDMA mediating the release of neurochemicals, BDNF, and growth factors. This modification can initiate enhanced cognition and behavior or support treatments in neurologic and psychiatric conditions. However, such promising positive effects probably appear only under specific conditions that need to be carefully controlled and evaluated.

The acute interactions of one session of exercise and tDCS on cognition have not been empirically addressed so far and should be systematically and experimentally investigated in the future. Special emphasis should determine whether there is a dose-response relationship in terms of exercise and tDCS intensity and duration, stimulation area and polarity, and whether the time course between exercise and stimulation could interact on several levels of the healthy and the impaired human brain. If synergistic or additive effects from acute combined effects can be experimentally observed in the cognitive or in the motor domain, future interventions may benefit from synergies between both techniques.

## Author Contributions

All authors provided substantial contributions to the work. FS drafted the manuscript. FF and NP revised it critically.

## Conflict of Interest Statement

The authors declare that the research was conducted in the absence of any commercial or financial relationships that could be construed as a potential conflict of interest.
